# Vacuum-Assisted Closure Therapy Following Mesh Removal Due to Multidrug-Resistant Pseudomonas aeruginosa Infection in a High-Risk, Multimorbid, Hernia Repair Patient: A Case Report and Literature Review

**DOI:** 10.7759/cureus.82492

**Published:** 2025-04-18

**Authors:** Christina Thankam Jijy, Aleena Anna Jijy, Martin Karamanliev, Meri Shoshkova., Harrie Toms John

**Affiliations:** 1 Surgical Oncology, UMHAT (University Multiprofile Hospital for Active Treatment) “Dr. Georgi Stranski” University Hospital, Pleven, BGR; 2 Critical Care Medicine, Epsom and St. Helier University Hospitals NHS Trust, London, GBR

**Keywords:** glucose intolerance, hernioplasty, infection, mesh, multi-drug resistant bacteria, multimorbid, vac therapy

## Abstract

Mesh infection is a significant complication after hernia surgery and is associated with increased morbidity, reoperation rates, and impaired quality of life. Risk factors include chronic obstructive pulmonary disease (COPD), obesity, diabetes, smoking, and advanced age. We present a 49-year-old obese woman with poorly controlled type 2 diabetes mellitus and multiple significant comorbidities, including congestive heart failure, permanent atrial fibrillation, chronic kidney disease, dyslipidemia, gout, bronchiectasis, and a history of multiple abdominal surgeries, who developed a postoperative mesh infection following onlay hernioplasty. Wound cultures revealed Pseudomonas aeruginosa with specific antibiotic sensitivities. Management involved mesh removal, extensive debridement, targeted antibiotic therapy, and vacuum-assisted closure (VAC) therapy followed by abdominoplasty, which led to complete wound healing. Despite successful infection management, the patient developed a recurrent hernia during follow-up. This case demonstrates the effectiveness of VAC therapy in managing infected mesh sites after hernia repair while highlighting the challenge of maintaining long-term hernia repair integrity following mesh removal. The case underscores the importance of balancing infection control with structural support in high-risk patients with multiple significant comorbidities.

## Introduction

Mesh implants represent the standard treatment for incisional hernia repair procedures [1], serving either to support redirected fascia or bridge damaged fascia. Despite surgical advances, mesh infection often necessitates explantation [2], with infection rates approaching 10% after ventral or incisional hernia mesh repair [3-5]. The problems associated with mesh infection are considerable, leading some surgeons to recommend against synthetic mesh when patient comorbidities, previous infections, or surgical circumstances indicate increased infection risk [6]. Patient-related risk factors, such as obesity, diabetes mellitus, smoking, cardiovascular disease, chronic kidney disease, and advanced age, significantly increase infection risk in the context of evolving microbial resistance.

Vacuum-assisted closure (VAC) therapy, introduced by Argenta and Morykwas [7], has revolutionized complex wound management. Implementation involves debridement, hemostasis, sterile foam application with a fenestrated tube, and airtight sealing, creating negative pressure (50-125 mmHg) with dressings changed every three days [8]. This therapy reduces edema and bacterial levels while enhancing tissue perfusion and granulation, making it suitable for complex, non-healing wounds [9] and offering a superior alternative to traditional dressings with decreased morbidity, costs, and hospital stays [10].

While vacuum-assisted closure (VAC) therapy has been reported as an effective adjunct in managing mesh infections, especially in postoperative wounds, literature is sparse on its application in patients with extensive comorbidity burden with conditions that complicate both the wound healing process and antibiotic pharmacodynamics. Moreover, data on VAC therapy outcomes in infections involving multidrug-resistant Pseudomonas aeruginosa remain limited. This case report aims to illustrate the application of VAC therapy in treating mesh infection following hernia repair and to evaluate its efficacy in promoting wound healing in a patient with multiple severe comorbidities and risk factors for poor surgical outcomes, while also discussing the challenges of hernia recurrence after mesh removal.

## Case presentation

A 49-year-old woman presented with signs of postoperative wound infection following hernioplasty with mesh placement (onlay technique) performed two weeks prior. Her medical history was significant for obesity (BMI 34.2 kg/m²), poorly controlled type 2 diabetes mellitus (HbA1c 9.8%), and a 10 pack-year smoking history. Additionally, the patient had congestive heart failure (CHF), permanent atrial fibrillation, chronic kidney disease (CKD), dyslipidemia, gout, bronchiectasis, and chronic calculous cholecystitis. Her surgical history included laparoscopic cholecystectomy, ventral hernia repair with herniotomy and hernioplasty due to incisional hernia, and hystero adnexectomy with herniotomy and hernioplasty for ovarian endometriosis with midline incision.

The patient reported a non-healing surgical wound characterized by increasing pain, pronounced erythema, and copious cloudy, whitish purulent discharge from the abdominal incision site for the past two days. She additionally experienced general malaise and anorexia but remained afebrile at presentation.

Physical examination revealed a hypersthenic habitus, irregular heart rhythm consistent with atrial fibrillation, clear lung fields, and a distended yet non-tender abdomen without peritoneal signs. The surgical incision showed extensive erythema surrounding the wound edges, visible purulent discharge around the sutures and from a cutaneous fistula, and exposed surgical mesh from the prior hernioplasty. Imaging was deferred given the overt clinical presentation of mesh exposure and purulent drainage, which established the diagnosis. Surgical exploration was prioritized over radiologic assessment.

Laboratory investigations, as outlined in Table 1, showed elevated markers of systemic inflammation, including high C-reactive protein (CRP), increased red cell distribution width (RDW), elevated direct bilirubin, and significant hyperglycemia, all suggestive of an advanced infectious process. Wound cultures were obtained, which later grew multidrug-resistant Pseudomonas aeruginosa.

**Table 1:** Laboratory and vital parameters overview of the patient MCV: mean corpuscular volume; MCH: mean corpuscular hemoglobin; MCHC: Mean corpuscular hemoglobin concentration; RDW: red cell distribution width; LDH: lactate dehydrogenase; ALT: alanine aminotransferase; AST: aspartate aminotransferase; aPTT: activated partial thromboplastin time

Parameter	Result	Unit	Reference Range
Vital Signs			
Blood Pressure (BP)	120/80	mmHg	~120/80
Heart Rate	110	bpm	60–100
Blood Count			
Leukocytes	9.43	×10⁹/L	3.5–10.5
Erythrocytes	4.65	×10¹²/L	3.75–5.3
Platelets	307.0	×10⁹/L	130–360
Hemoglobin	126.0	g/L	120–160
Hematocrit	0.39	L/L	0.38–0.48
MCV	84.0	fL	82–96
MCH	27.1	pg	27–33
MCHC	323.0	g/L	300–360
RDW	17.5	%	11.5–14.5
CRP	91.62	mg/L	0–5
Total Serum Protein	74.6	g/L	66–87
Renal Function			
Urea	4.8	mmol/L	2.8–8.1
White Cell Differential			
Lymphocytes	18.4	%	20–48
Monocytes	8.1	%	1–11
Neutrophils	70.8	%	40–76
Eosinophils	0.8	%	0–6
Basophils	0.5	%	0–1.5
Enzymes & Proteins			
LDH	277.0	U/L	240–480
Serum Albumin	38.5	g/L	35–52
Fibrinogen	6.53	g/L	2–4
Liver Function			
Direct Bilirubin	12.6	µmol/L	0–5
ALAT (ALT)	15.8	U/L	0–40
ASAT (AST)	18.7	U/L	0–40
Electrolytes			
Sodium (Na)	133	mmol/L	135–155
Potassium (K)	4.2	mmol/L	3.5–5.3
Chloride	93	mmol/L	98–108
Coagulation Tests			
aPTT (seconds)	24.6	sec	~24–36 (lab-specific)
aPTT Ratio	0.95	ratio	0.8–1.2

**Figure 1:**
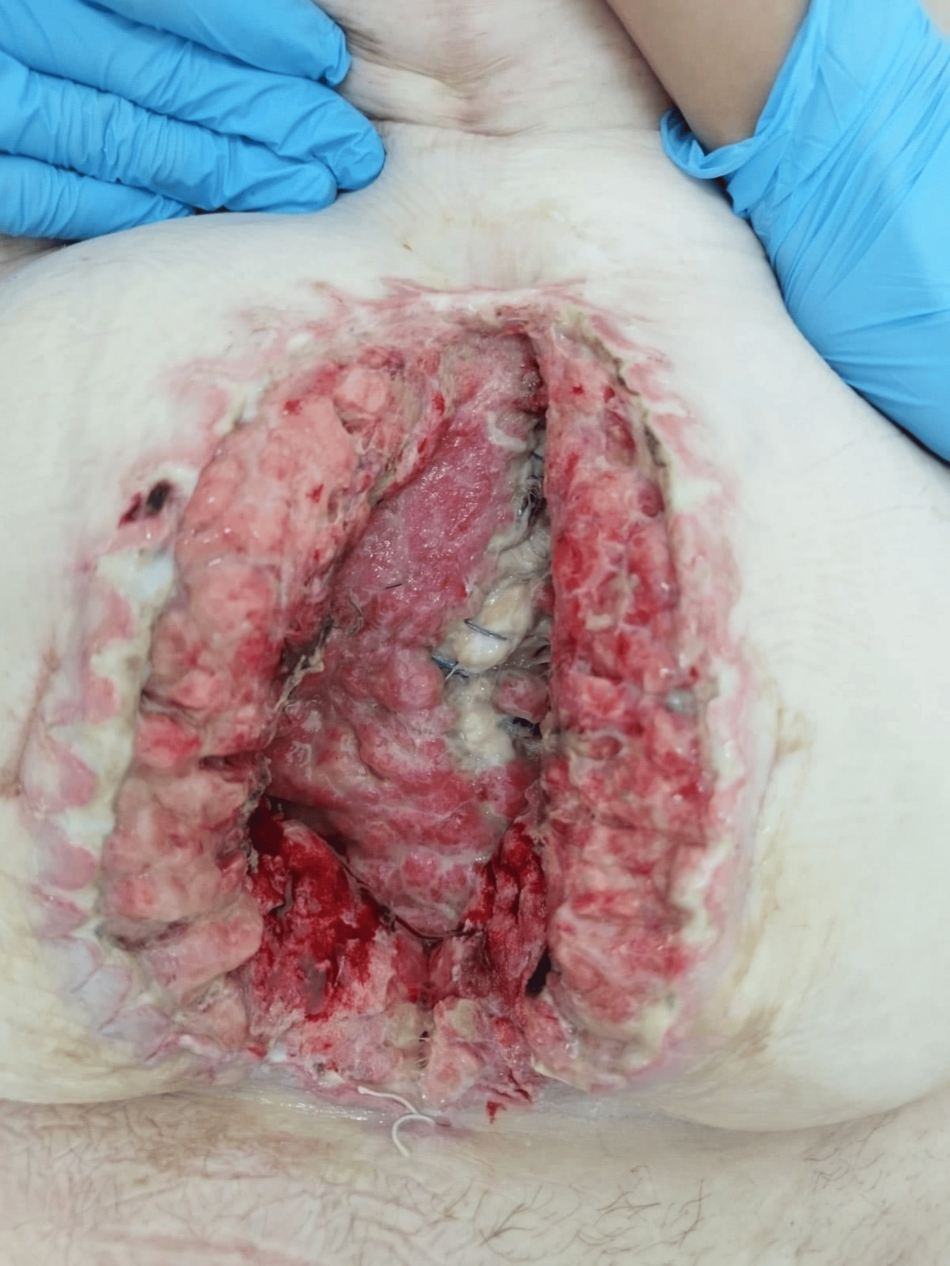
Surgically extracted infected mesh from the abdominal wall at the hernia repair site, prior to surgical debridement and the initiation of vacuum-assisted closure (VAC) therapy

**Figure 2:**
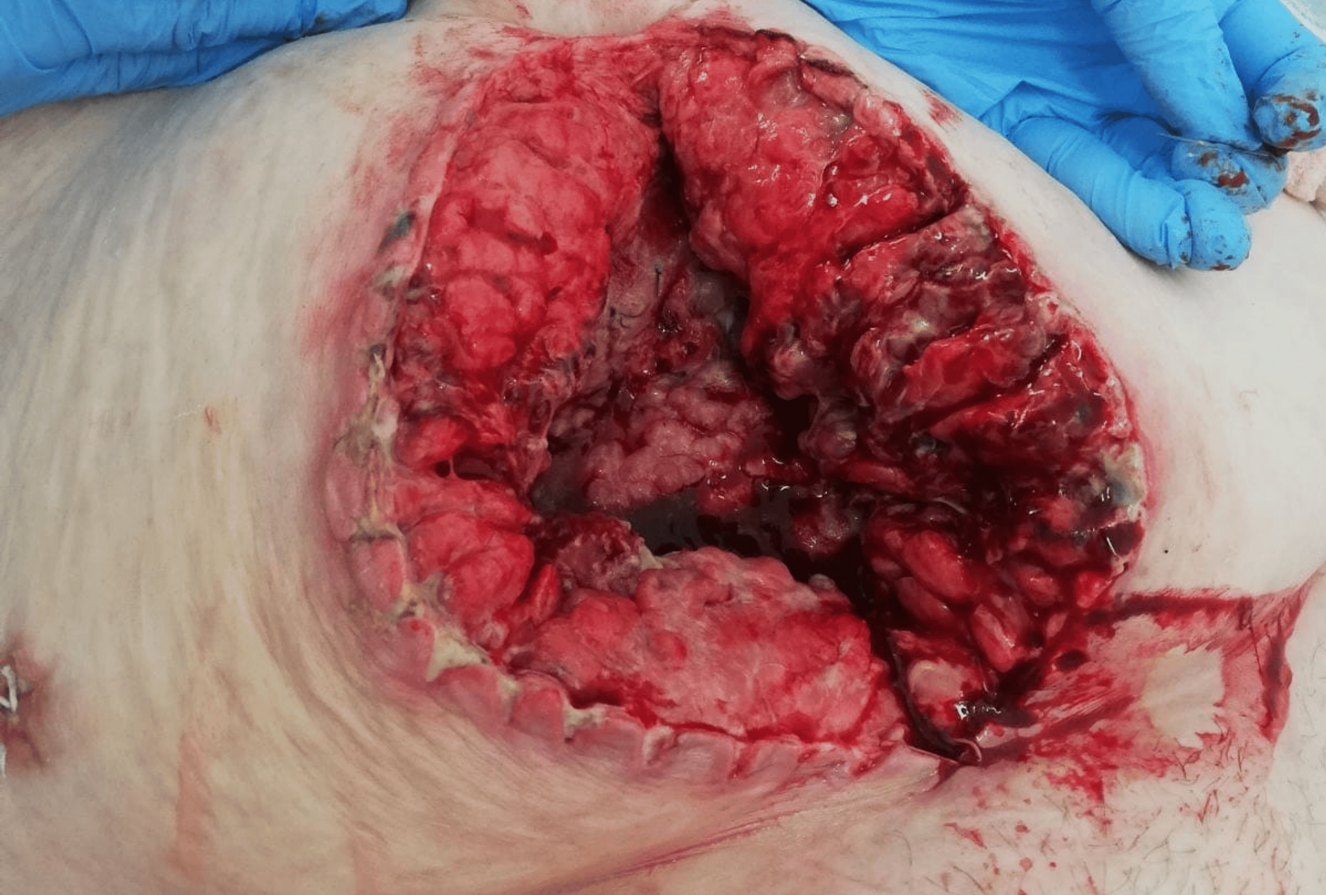
View of the hernia repair site following thorough surgical debridement, demonstrating cleaner wound margins and preparation for VAC therapy application.

**Figure 3:**
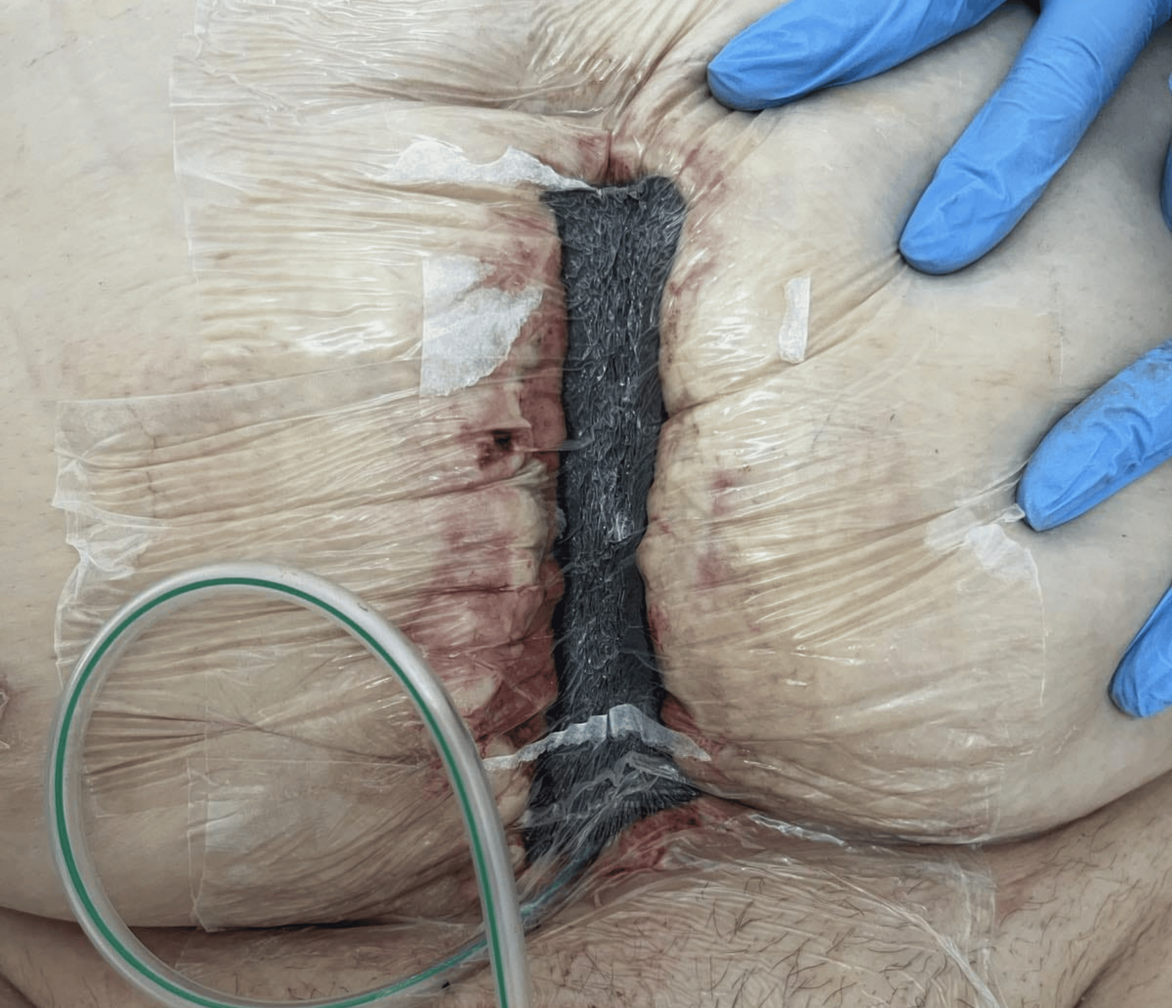
Application of vacuum-assisted closure (VAC) therapy over the debrided hernia repair site, showing placement of foam dressing and tubing to promote wound healing and infection control

**Figure 4:**
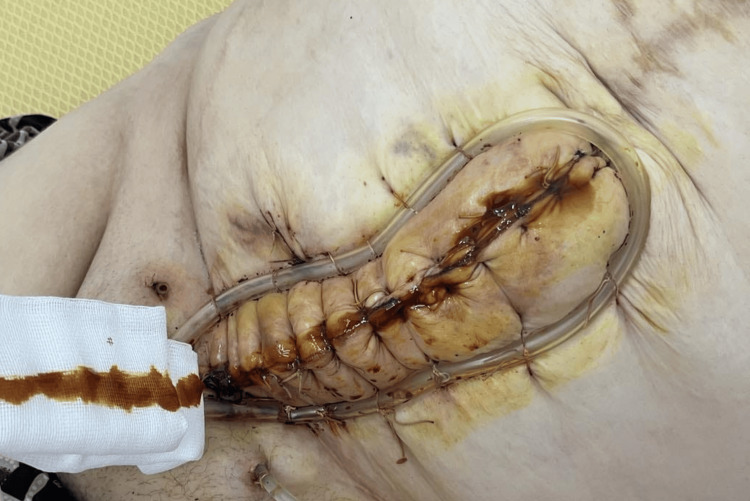
Continued management of the abdominal wound with vacuum-assisted closure (VAC) therapy, highlighting wound bed improvement.

A diagnosis of abdominal wall phlegmon secondary to infected mesh hernioplasty was established. After careful consideration of the patient's extensive comorbidities and surgical risk assessment by a multidisciplinary team, including a cardiology and nephrology consultation, the patient underwent urgent surgical intervention, which included the complete removal of the infected polypropylene mesh (Figure 1) followed by aggressive debridement of necrotic and infected tissue (Figure 2). A thorough subcutaneous lavage was performed with 3L of normal saline containing gentamicin (80 mg/L). Placement of a VAC dressing was initiated with continuous negative pressure at 180 mmHg (Figures 3, 4).

The patient was initially started on empiric intravenous meropenem due to the high risk of gram-negative and potentially resistant organisms in the postoperative setting. Once the wound culture and sensitivity results became available and demonstrated multidrug-resistant Pseudomonas aeruginosa resistant to meropenem, as mentioned in Table 2, the antibiotic regimen was appropriately de-escalated. Based on culture sensitivities, the patient received a combination antibiotic therapy with intravenous amikacin (15 mg/kg daily, with therapeutic drug monitoring) and intravenous colistin (9 million units loading dose followed by 4.5 million units every 12 hours) for synergistic effect against the multidrug-resistant Pseudomonas aeruginosa. Due to the patient's pre-existing chronic kidney disease, renal function was monitored daily with dosage adjustments based on calculated creatinine clearance. Cardiology management included the continuation of anticoagulation for atrial fibrillation with bridging therapy and appropriate heart failure management.

**Table 2:** Antibiotic susceptibility report for Pseudomonas aeruginosa from wound culture

Antibiotic	Sensitivity
Amikacin	Sensitive (S)
Cefepime	Resistant (R)
Ceftazidime	Resistant (R)
Ciprofloxacin	Resistant (R)
Colistin	Sensitive (S)
Gentamicin	Not specified (—)
Imipenem	Resistant (R)
Levofloxacin	Resistant (R)
Meropenem	Resistant (R)
Piperacillin	Resistant (R)
Piperacillin-tazobactam	Resistant (R)
Tobramycin	Resistant (R)
Cefoperazone/sulbactam	Not specified (—)

The VAC dressing was changed every 72 hours for 14 days, during which progressive improvement in wound granulation was observed. Blood glucose management was optimized with an insulin regimen, achieving target values between 110-140 mg/dL. Fluid status was carefully monitored due to the patient's heart failure, with parameters adjusted during VAC therapy to prevent volume overload.

**Figure 5:**
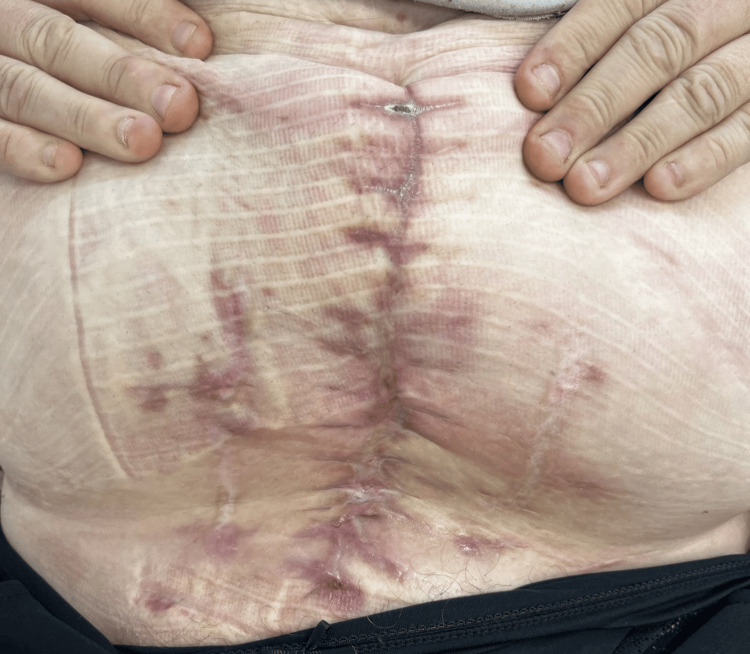
Post-treatment image of the abdominal wound demonstrating complete epithelialization and wound closure following successful completion of vacuum-assisted closure (VAC) therapy and secondary abdominoplasty

After achieving adequate granulation tissue formation, secondary abdominoplasty was performed to achieve wound closure without tension. The procedure duration was minimized to reduce cardiac risk, and postoperative monitoring in an intermediate care unit was implemented due to the patient's cardiac and renal comorbidities. Postoperative recovery was favorable, with marked clinical improvement and resolution of infection (Figure 5). The patient was discharged on postoperative day 5 after the abdominoplasty in good general condition, with plans for outpatient follow-up and ongoing glycemic management as well as continued care for her cardiac, renal, and metabolic conditions.

At the three-month follow-up, the wound had completely healed with acceptable cosmetic results. However, the patient reported increasing abdominal discomfort and bulging at the surgical site. Physical examination confirmed the development of a recurrent incisional hernia. Given the recent history of infection and the patient's extensive comorbidities, including cardiac and renal disease, a conservative approach was initially recommended with abdominal binder support and weight loss counseling.

## Discussion

Since Morykwas and Argenta introduced VAC therapy in the early 1990s, negative pressure wound therapy has transformed complex wound treatment [11,12]. Their animal experiments demonstrated that topical negative pressure therapy enhances local blood flow, accelerates granulation tissue formation, promotes bacterial clearance, and improves flap survival.

VAC therapy activates multiple simultaneous processes for wound healing. Subjecting affected tissue to multiple sub-atmospheric pressure cycles triggers wound cell cytoskeleton activation, which starts cellular sequences that promote tissue expansion [8]. Vacuum therapy eliminates extra fluid and exudate from the tissue while simultaneously reducing swelling and delivering better circulation, along with promoting new blood vessel growth and granulation tissue development. The combination of effects generates conditions suitable for effective wound healing.

The clinical use of VAC therapy has proven effective for treating various wounds, including infected surgical wounds, along with traumatic wounds, pressure ulcers, and exposed bones and hardware, as well as diabetic foot ulcers and venous stasis ulcers. The mechanisms of action include exudate removal, wound edge approximation, microenvironment alteration, and promotion of granulation tissue formation and angiogenesis [13]. This therapeutic approach plays a vital role in postoperative mesh infection management following hernia repairs, which can be observed in the presented clinical example.

Our case presents several features of clinical significance. First, the causative organism was a multidrug-resistant strain of Pseudomonas aeruginosa, sensitive only to amikacin and colistin. Infections caused by Pseudomonas bacteria prove troublesome for prosthetic material because these microorganisms develop biofilms that protect bacteria from immune responses while obstructing medication penetration. The resistance pattern necessitated the use of potentially toxic antibiotics with close monitoring, particularly challenging given the patient's pre-existing chronic kidney disease.

The patient’s extensive comorbidities significantly influenced both the development of infection and the selection of a treatment strategy. CHF and atrial fibrillation alongside metabolic disorders from obesity through diabetes, dyslipidemia, and gout produced a sequence of poor tissue perfusion with compromised immunity and reduced tissue oxygen levels. Multiple previous abdominal surgeries, together with mesh placement, made the surgical field more complex while potentially leading to the current complication.

Reports from previous research demonstrate that VAC therapy effectively treats mesh infections. Tawes and Najarian documented four patients who got abdominal wall hernia mesh infections following surgery and received VAC treatment after draining sites until their wounds grew enough granulation tissue. However, their study did not provide specific information about the long-term possibility of hernia recurrence after mesh removal or the application of this technique in patients with multiple severe comorbidities [14].

Our specific case demonstrates how patient characteristics created conditions that fostered both mesh infection and recurrent hernia problems. The combination of obesity and diabetes mellitus creates conditions for surgical site infections because it blocks adequate tissue blood flow, weakens immune defenses, and delays healing. The patient's history of smoking aggravated both the problem of mesh infection and tissue oxygenation issues affecting healing potential. The cardiovascular comorbidities, particularly CHF and atrial fibrillation, further impaired peripheral tissue perfusion, while CKD impacted both immune function and medication clearance. These comorbidities significantly elevated the patient's surgical risk profile and required careful multidisciplinary management.

The decision to remove the infected mesh rather than attempt mesh salvage was based on the extent of infection, the presence of a cutaneous fistula with mesh exposure, and the isolation of multidrug-resistant Pseudomonas aeruginosa. While some studies suggest that large-pore meshes may be salvaged with topical negative pressure therapy [3], our patient's clinical presentation and the nature of the infecting organism warranted complete mesh removal to ensure adequate source control.

The development of a recurrent hernia following mesh removal highlights one of the principal challenges in managing mesh infections. While removing the infected mesh is often necessary to control infection, it significantly increases the risk of hernia recurrence. The systematic review conducted by Bueno-Lledó et al. demonstrated an infection-related mesh explantation recurrence range between 21.9% and 51.1% [15]. The recurrence in our patient was consistent with these findings and underscores the importance of considering both infection control and long-term structural support in the management plan, particularly challenging in patients with multiple risk factors for both infection and recurrence.

Infection was successfully managed through a combined approach of mesh removal, targeted antibiotic therapy based on sensitivity results, and vacuum-assisted wound therapy followed by delayed surgical reconstruction. The VAC therapy prepared the wound bed by removing invasive materials and managing swelling while promoting growth of new tissue that led to successful abdominoplasty secondary closure. However, this approach did not prevent hernia recurrence.

Future management options for this patient include delayed repair with biological mesh once the infection has completely resolved and modifiable risk factors have been addressed. Biological meshes demonstrate properties that help them succeed in previously infected fields through infection resistance and tissue integration, yet they lead to more recurrences than synthetic meshes [16]. For our patient, optimization of cardiovascular status, improved glycemic control, and stabilization of renal function will be critical before attempting any further reconstructive procedures.

This case underscores the critical importance of recognizing patient-specific risk factors for mesh infection and hernia recurrence, particularly in the setting of complex comorbidities. To our knowledge, few published reports have detailed wound closure success in a patient presenting with this constellation of risk factors, such as CHF, permanent AF, CKD, and morbid obesity. What distinguishes this case is not only the presence of a multi-drug resistant organism, a known challenge in mesh infections but also the successful use of VAC therapy in a patient with exceptional systemic vulnerability. It highlights the need for appropriate cultures with sensitivity testing to guide antibiotic therapy and the implementation of prompt, aggressive management strategies. Moreover, it demonstrates the utility of VAC therapy as an adjunctive measure in managing complex surgical site infections involving resistant organisms, while candidly illustrating its limitations in preventing hernia recurrence after mesh removal, especially in multimorbid patients lacking structural support.

## Conclusions

This case demonstrates how VAC therapy successfully controlled mesh infection after hernioplasty by treating a high-risk patient with multiple severe comorbidities, including congestive heart failure, atrial fibrillation, chronic kidney disease, and metabolic disorders. Follow-up treatment, which began with mesh removal, then included debridement and culture-based antibiotic selection, followed by VAC therapy and delayed surgical closure, permitted successful wound healing, though it developed into recurrent hernia formation. This case shows the difficulty of treating mesh infections properly without compromising abdominal wall construct stability. The onlay mesh position has a higher risk of infection than the sublay retromuscular position. The beneficial effects of VAC therapy for treating surgical site infections with prosthetic materials should not substitute for the biomechanical roles of mesh reinforcement. This case also underscores the critical importance of multidisciplinary management in patients with complex comorbidity profiles. Surgical decisions must be made with careful consideration of the patient's cardiac status, renal function, metabolic control, and prior surgical history. In such complex cases, the primary goal must be infection control and patient survival, even when this may come at the expense of definitive hernia repair.

Future studies should create strategies to stop mesh removal infections from causing hernia recurrence by determining suitable mesh materials for reconstruction after infection and investigating the most effective timing for delayed repairs, as well as new methods to support the abdominal wall, particularly in patients with multiple comorbidities that increase both infection risk and surgical complexity.
